# EXPLORING IKIGAI AND LIVING WELL WITH DEMENTIA AMONG JAPANESE CANADIANS: A NARRATIVE AND ARTS-BASED RESEARCH STUDY

**DOI:** 10.1093/geroni/igae098.2129

**Published:** 2024-12-31

**Authors:** Hiro Ito, Susan Cox, Alison Phinney, Anita Ho

**Affiliations:** University of British Columbia, Vancouver, British Columbia, Canada; University of British Columbia, Vancouver, British Columbia, Canada; University of British Columbia, Vancouver, British Columbia, Canada; University of British Columbia, Canada

## Abstract

Like many ethnocultural minority populations, Japanese Canadians have been underrepresented in research pertaining to experiences of living with dementia. Representation of how diverse populations experience living with dementia is necessary to further scholarship on the intersections of dementia and culture. Thus, we aim to explore how Japanese Canadians live well with dementia through the conceptual lens of ikigai, a Japanese wellbeing construct. In this two-phase study, we took a relational approach and recruited 4 people living with dementia and 3 of their care partners from the Japanese Canadian community. In the first phase, we conducted individual and dyadic narrative interviews to understand experiences of living well with dementia. In the second phase, we gathered in a group art-making workshop to enable participants to identify and express what they wanted others to know about how they live well with dementia. To ensure that individuals could participate meaningfully in their preferred language, the study was conducted in English and Japanese. Audio recordings from both phases were transcribed and analyzed through narrative inquiry. Through our study, we identified several ways Japanese Canadians maintained their ikigai to live well with dementia, including notions of balance, continuity, and gratitude. Our findings offer insights into the integral role of culture in living well with dementia, as well as the value of culturally nuanced and inclusive research.

